# Management of tinnitus in English NHS audiology departments: an evaluation of current practice

**DOI:** 10.1111/j.1365-2753.2010.01566.x

**Published:** 2012-04

**Authors:** Derek J Hoare, Phillip E Gander, Luke Collins, Sandra Smith, Deborah A Hall

**Affiliations:** 1 Research FellowNottingham, UK; 2 Research AssociateNottingham, UK; 3 Lead Scientist, NIHR National Biomedical Research Unit in Hearing, School of Clinical Sciences, The University of NottinghamNottingham, UK

**Keywords:** good practice guidelines, hearing therapist, outcome measure

## Abstract

**Rationale, aim and objective:**

In 2009, the UK Department of Health formalized recommended National Health Service practices for the management of tinnitus from primary care onwards. It is timely therefore to evaluate the perceived practicality, utility and impact of those guidelines in the context of current practice.

**Methods:**

We surveyed current practice by posting a 36-item questionnaire to all audiology and hearing therapy staff that we were able to identify as being involved in tinnitus patient care in England.

**Results:**

In total, 138 out of 351 clinicians responded (39% response rate). The findings indicate a consensus opinion that management should be tailored to individual symptom profiles but that there is little standardization of assessment procedures or tools in use.

**Conclusions:**

While the lack of standardized practice might provide flexibility to meet local demand, it has drawbacks. It makes it difficult to ascertain key standards of best practice, it complicates the process of clinical audit, it implies unequal patient access to care, and it limits the implementation of translational research outcomes. We recommend that core elements of practice should be standardized, including use of a validated tinnitus questionnaires and an agreed pathway for decision making to better understand the rationale for management strategies offered.

## Introduction

Tinnitus aurium, the sensation of noise in the absence of an external sound, is thought to be related to changes in the activity or connectivity of the auditory cortex [1], and is primarily associated with noise exposure [2] and ageing [3]. It affects an estimated 10–15% of people in the UK at some point in their lives [4], and affects up to one in three older adults [5] of which a substantial proportion access medical and audiological services. For these people, tinnitus has a detrimental impact on their daily life [6] and can co-occur with anxiety, depression and suicidal tendencies [7]. For example, in a sample of 92 adults, Adoga *et al*. [8] reported a 17% co-morbidity of tinnitus with depression. There is also a high incidence of sleep disturbance associated with tinnitus [3], and for many patients, underlying hearing loss is also a significant factor in the perceived distress or discomfort of tinnitus [9, 10]. In an analysis of data from 1240 patients, Shao *et al*. [11] found that 76% had sensorineural hearing loss and that 90% reported some form of psychological adverse reaction, either irritability, insomnia or an inability to concentrate.

The absence of an effective cure for tinnitus leaves the patient with the prospect of adjusting to this abnormal sensation, as well as to any psychological co-morbidity. As such, this patient population can present significant challenges to the clinicians who consult them. As tinnitus is often associated with hearing loss, many patients follow a referral path to Audiology for management. In 2009, the UK Department of Health (DH) issued a Good Practice Guide (GPG) [12] for the commissioning of tinnitus services and for the management of tinnitus in primary care (General Practitioners), local tinnitus services (audiologists and hearing therapists), specialist centres [multi-disciplinary teams (MDT) to include audiologists, hearing therapists, ear, nose and throat (ENT) surgeons, audiovestibular physicians and clinical psychologists] and supra-specialist centres (MDT that can additionally offer complex audiological assessments, neurosurgical intervention and radiotherapy). The GPG forms part of a continued drive to realize the vision for service quality set out in the DH document ‘Improving Access to Audiology Services in England’[13].

While specific, the GPG cites limited primary or high-level evidence (i.e. randomized-controlled trials or meta-analyses) underpinning the tinnitus management practices that it recommends for the National Health Service (NHS). Such evidence is collated in the NHS Annual Evidence Update (http://www.library.nhs.uk/ENT/) which provides a comprehensive summary of all tinnitus-related publications each year. Examination of this resource indicates mixed evidence to support the clinical efficacy of the different management strategies. For example, Moffatt *et al*. [14] reported that hearing-aid fitting has limited effects on the perception of tinnitus. However, Trotter & Donaldson [15] with a caseload of 1440 tinnitus patients who had received hearing aids, found significantly improved visual analogue scale (VAS) scores of tinnitus percept in 68% of cases. Similarly, Lugli *et al*. [16] found that sound treatment was beneficial, but Hesser *et al*. [17] found that sound generators can be associated with increased disability in individuals with tinnitus. In terms of ‘talking therapies’ Zeng *et al*. [18] found that 75% of tinnitus patients were successfully managed by educational counselling alone. Cognitive Behavioural Therapy (CBT) is also reported to be an effective management strategy [19] including as a group or internet-based therapy [20]. Tinnitus Retraining Therapy (TRT) is reported as being effective after 1 month [21] and was found to reduce disability associated with chronic tinnitus for up to 18 months after the therapy had ended [22]. Sakashita *et al*. [23] found that 70% of tinnitus patients improved with TRT but that it was least effective for patients with psychological or psychiatric problems. Combinations of therapies also show benefits. For example, Gudex *et al*. [24] reported significant improvement when management involved a combination of counselling and a listening device.

Given the context of specific DH guidelines, yet limited high-quality evidence for the efficacy of different management strategies, our objective here was to assess how tinnitus is managed in current clinical practice, to evaluate how this aligns with DH recommendations, and to explore the perceived difficulties or barriers to effective management. To do this we posed questions on tinnitus assessment, management, outcome, clinical skill and resources, to clinicians in audiology departments across England. In a separate report we discuss the opinions of clinicians on the broader current health care system related to tinnitus management in the NHS.

## Methods

### Questionnaire development

Generation of a 36-item questionnaire followed a systematic approach to survey design based on Kelley *et al*. [25] and Burns *et al*. [26]. In the first step, authors identified general items for inclusion in the questionnaire which were grouped according to 10 emergent topics: the 18-week pathway, DH GPG, the referral process, specialist training, departmental staffing, resource management, assessment, treatment, outcome measurement and support networks. The choice of these topics was informed by NHS literature and online resources, scientific papers and anecdotal comments from clinical colleagues in audiology and ENT. Researchers then formulated a large number of potential questions within each topic based on all the items categorized.

Topics formed the basis for discussion at a clinical focus group between lead researchers, four audiologists and one ENT specialist. Participating clinicians worked within the South West, West Midlands, North East, Yorkshire and the Humber Strategic Health Authorities (SHAs) and were attending the British Tinnitus Association (BTA) 2009 conference. An audiovestibular physician from the North West SHA also participated in a one-to-one discussion with one of the research team. Discussion focused on identifying relevant items for inclusion in the questionnaire, and on highlighting weak or redundant items. This information was used to compile a set of questions and response options. To minimize potential bias, an expert in qualitative research advised on the wording of several questions (K. Levine, personal communication). The first version of the questionnaire was piloted at a second clinical focus group held between the same researchers, six audiologists and two ENT specialists from Nottingham University Hospitals NHS Trust. The purpose of this focus group was to assess the construct validity of the questions and response options (are clinicians assigning the same meaning to the text?) and the face validity of the questions (are clinicians answering the question we wish to ask?) [26]. Feedback was used to revise several items. The final version of the questionnaire included 24 ‘tick box’ questions which provided a list of response options and an ‘other’ category, and 12 open questions which required a written response. Open questions provide a good way of eliciting personal opinions and to identify how strongly attitudes are held or not. Respondents were additionally asked to identify their job role. A list of the questions related to this report is given in Appendix 1.

### Distribution

A database of NHS Trust audiology departments in England was compiled from mailing lists provided by the BTA and the Royal National Institute for Deaf people (RNID). The sampling method was purposive. Every audiology department on the database was contacted by telephone or email to identify names of individuals who either manage or directly provide clinical services for people with tinnitus. In total, 351 individuals were identified. In November 2009, the questionnaire was mailed to all 351 individuals with a return envelope and a cover letter describing the purpose of the questionnaire, completion instructions, and details of a prize draw for all those responding within 8 weeks of the initial mailing. A reminder letter and further copy of the questionnaire was sent after 10 weeks. Although we identified our target population by name, the questionnaires were returned anonymously.

### Data collection and analysis

All responses were entered into a Microsoft Access database. Descriptive statistics and analyses were performed in PAST (version 2.02 [27]). Responses to open questions (free-text answers) required appropriate qualitative analysis and so were subjected to a thematic content analysis [28].

### Thematic analysis of free-text responses

Thematic analysis involves coding and categorizing sections of written or transcribed text based on the themes of the text. It is an analytic method that is widely used in qualitative research [28–30]. A protocol for the conduct of thematic analysis was based on Braun & Clarke [31], and was followed by all authors. To improve reliability, responses to open question were analyzed independently by a pair of authors. The process had five stages. (i) The first stage was a familiarization, or immersion process, where each author read and reread all of the responses to a question. (ii) With that specific question in mind, the next stage was an active reading process where responses that appropriately addressed the question were selected. At the same time the reader looked for recurring themes or concepts running through the responses. (iii) At the next stage, initial codes were generated. Codes identify a feature of the individual response that appears relevant to the analyst [28] and here referred to the most basic meaningful element of the text. Critically, the chosen segment of each response had to maintain the meaning and context intended by the respondent. (iv) Codes that were considered to be equivalent were grouped under ‘proposed themes’. Only after this stage, did the two authors meet to agree ‘codes’ and ‘proposed themes’, revisiting the full dataset to confirm the likeness of codes within a theme and the distinctiveness of codes classified under different themes. (v) The final stage involved a review of the proposed themes where all five authors reached a consensus on the suitability of each theme.

## Results

We received 138 responses (39% response rate) from 42 hearing therapists, 80 audiologists, 1 clinical psychologist, and 15 respondents who did not indicate their job role. Twenty-nine responses came from clinicians working in local audiology services and the other 109 were from clinicians working in either specialist or supra-specialist audiology centres (as defined by the DH [12]). Unless otherwise stated, response rates (in percentages) were calculated from the total number of 138 responses. The following sections consider appointment structure, diagnostic assessment, management, outcome assessment, determinants of a successful outcome, and clinical resourcing. Where appropriate, responses are subdivided by job role, audiologist (*n* = 48), hearing therapist (*n* = 42), manager (*n* = 32), or by type of department, local tinnitus service (*n* = 29) or specialist/supra-specialist centre (*n* = 109).

### Appointment structure

The GPG does not make specific recommendations about appointment structure. When asked about appointment times (Question i, Appendix 1) the average length of a tinnitus consultation was reported to be 1 hour for diagnostic assessment or management, or 1.25 hours for a combined appointment. The length of time allocated for diagnostic assessment varied from 15 to 150 minutes. Management and combined appointments were both reported as being allocated 45 to 150 minutes. Thereby, patients who receive combined appointments receive a mean 38% less contact time than patients whose consultations are separate. The duration of appointments did not differ significantly between audiologists and hearing therapists for diagnosis (*z* = 0.633, *P* = 0.53, Mann–Whitney *U*-test) or management (*z* = −1.715, *P* = 0.08, Mann–Whitney *U*-test). In response to Question ii (Appendix 1), only 11% of respondents would *not* offer a combined appointment. We did not ask about duration of appointments to evaluate outcome.

When asked about family involvement (Question iii, Appendix 1), most clinicians encouraged it at the diagnostic assessment (71%), management (71%) and outcome (52%) appointments. The degree of family involvement by audiologists or hearing therapists was not significantly different (55% vs. 57%, *P* = 0.319, Fisher's exact test). Highlighted as a good practice example by the DH [12], just 20% of all clinicians offered group therapy sessions. This differed significantly by job role, hearing therapists were more likely to offer group sessions (30% vs. 15%, *P* = 0.017, Fisher's exact test).

### Diagnostic assessment

The DH recommendations comprise audiological assessment (audiometry, reflexes, otoacoustic emissions), psychoacoustic tinnitus measures (minimum masking level, loudness), screening for anxiety and depression with use of the Beck Anxiety Inventory (BAI [32]), and the Beck Depression Inventory (BDI [32]) or the Hospital Anxiety and Depression Scale (HADS [33]), and a self-reported measure of tinnitus severity such as the Tinnitus Handicap Inventory (THI [34]) or the Goebel-Hiller Tinnitus Questionnaire (GHTQ [35]).

**Figure 1:**
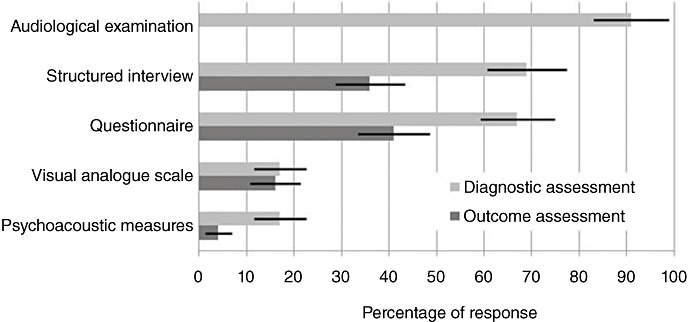
Histogram showing the use of different procedures for tinnitus diagnostic and outcome assessments. Responses indicate personal use of each procedure and not necessarily departmental level use. Responses are given as a percentage of total respondents (±95% confidence intervals).

**Figure 2:**
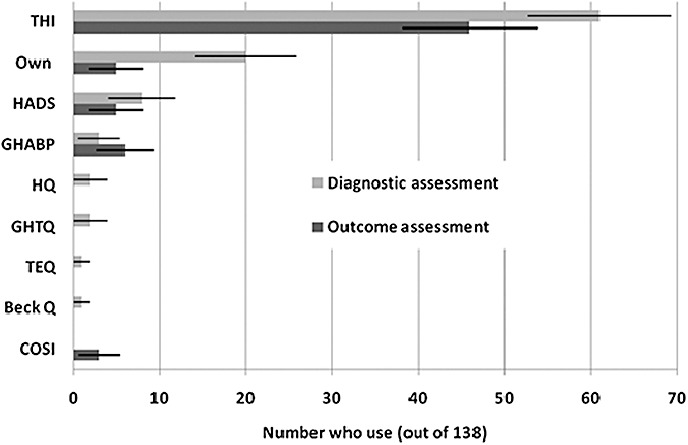
Histogram showing the use of different types of questionnaire in diagnostic (light bars) and outcome (dark bars) assessment stages. Responses are given as the number of respondents (±95% confidence intervals) who indicated that they use each questionnaire. THI, Tinnitus Handicap Inventory; Own, a self-styled questionnaire; HADS, Hospital Anxiety and Depression Scale; GHABP, Glasgow Hearing Aid Benefit Profile; HQ, Hyperacusis Questionnaire; GHTQ, Goebel-Hiller Tinnitus Questionnaire; TEQ, Tinnitus Effect Questionnaire; Beck Q, Beck Anxiety Inventory and Beck Depression Inventory; COSI, Client Oriented Scale of Improvement.

In current practice, the survey results indicate typical use of audiological measures and a self-reported measure of tinnitus severity (Fig. 1). In total, 91% of respondents routinely conduct an audiological examination (almost all conduct an audiogram, while only 2% reported that they routinely conduct otoacoustic emissions and reflexes). To assess tinnitus, 69% use a structured interview, 17% use a VAS, 17% use psychoacoustic measures of tinnitus pitch or loudness, and 67% use some form of questionnaire. The most common validated questionnaire was the THI, reported by 45% (Fig. 2). The THI is used equally by local services and specialist centres (38% vs. 46%, *P* = 0.352, Fisher's exact test). A total of 14% of respondents use a self-styled questionnaire. A minority (2%) use one of two other validated tinnitus questionnaires, the Tinnitus Effects Questionnaire [36] or the GHTQ. Other questionnaires in use are the Hyperacusis Questionnaire [37], the HADS questionnaire, the Glasgow Hearing Aid Benefit Profile [38] and the BAI and BDI, but by less than 10 respondents in all cases (see Fig. 2).

Although there was no significant difference between the type of diagnostic assessment used by hearing therapists and audiologists (for structured interview 68% vs. 64%, *P* = 0.427, Fisher's exact test, and for use of a validated questionnaire 61% vs. 50%, *P* = 0.052, Fisher's exact test), it was noted that management-level audiologists were significantly *less* likely to use a validated questionnaire for assessment than non-management level audiologists (25% vs. 65%, *P* < 0.001, Fisher's exact test).

When asked if the assessment procedure was standardized (Question vi, Appendix 1), 51% of respondents said that all clinicians within their department used the same assessment protocol. However, many were unsure (26%). Significantly more management level clinicians reported that assessment was standardized in their department than non-management clinicians (78% vs. 21%, *P* = 0.003, Fisher's exact test). This pattern indicates some degree of variability in the approach and by implication potential variability in the standard of care delivered to patients within the same department. If assessment is conducted in different ways by clinicians, it is conceivable that different approaches highlight different symptoms and different co-morbid factors and so subsequent management strategies might also differ.

When asked how standardized should the assessment procedure be (Question vii, Appendix 1), 25% of clinicians stated that it should be very standardized and 57% said that it should be standardized enough to provide a guide for junior or less experienced staff but should allow the clinician sufficient scope to address the needs of each tinnitus patient as an individual. The remaining 17% said it should not be standardized in any way, some of whom felt that any level of standardization of tinnitus assessment would be too restrictive.

### Tinnitus management

The GPG recommends tinnitus management with information/education, hearing aids, psychological support, relaxation therapy, CBT, sleep management, sound enrichment therapy, and TRT [12]. It is important to note here that the DH does not recommend TRT strictly according to the Jastreboff model [39], training for which is no longer funded by the NHS, but instead refers to any combination of management approaches that aim to habituate patients to their tinnitus (for one example of this ‘simplified TRT’, see Aazh *et al*. [40]). For clarity hereafter, the term ‘habituation therapies’ is used instead of TRT when referring to NHS practices.

**Figure 3:**
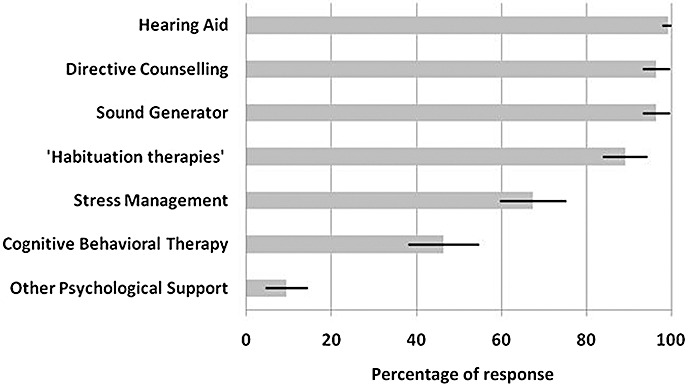
Management strategies employed in audiological departments. ‘Habituation therapies’ refers to any form of therapy that combines counselling and sound enrichment to promote habituation to tinnitus. ‘Other psychological support’ includes relaxation training or therapy, and acceptance and commitment therapy. Responses are given as a percentage of total respondents (±95% confidence intervals).

In response to the question of tinnitus management (Question viii, Appendix 1), a number of strategies were reported (Fig. 3). Almost all respondents offer hearing aids (99%), directive counselling (information and reassurance) (96%), sound generators (96%) and habituation therapies (89%). In total, 67% offer stress management, 46% offer some form of CBT, although when asked (Question ix, Appendix 1), only one third reported having the option to refer to a psychologist (Fig. 3). Nine percent offer other psychological support such as relaxation, and acceptance and commitment therapy. However, all respondents stated that they offer combinations of the above. In particular, all respondents who provided hearing aids or sound generators also offer directive counselling.

**Figure 4:**
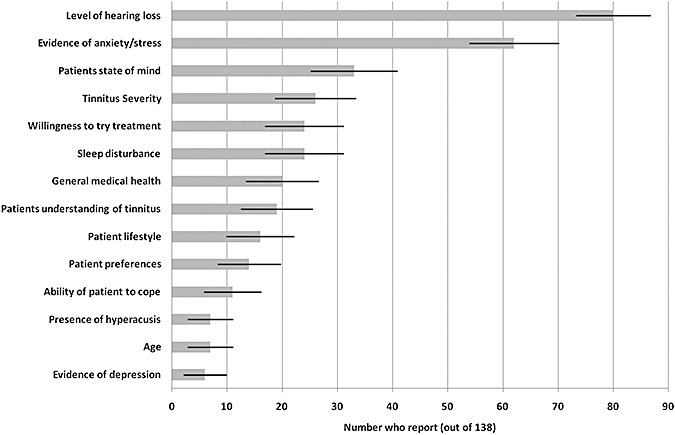
Histogram showing the reported factors that influence the choice of tinnitus management strategy. Responses are given as the number of respondents (±95% confidence intervals) who identified each factor.

A large number of factors were reported to influence the choice of management strategy (Question x, Appendix 1). These are illustrated in Fig. 4. The main influencing factor was hearing loss (80%). This concurs with the 99% who reported hearing-aid fitting as a management strategy. Other major factors included the presence of anxiety or stress, the patient's state of mind, and the severity of their tinnitus.

When asked specifically about hearing aid fitting (Question xi, Appendix 1), 47% of respondents reported using different criteria when offering a hearing aid to a patient with tinnitus than without. For example, some clinicians are more likely to fit a hearing aid for a milder sensorineural hearing loss, and the choice of device would be an open fit.

### Outcome assessment

The GPG does not specifically recommend assessing therapeutic outcomes. This is an important omission given that tinnitus management often constitutes a complex intervention, that is, there are several interacting components in the care that is delivered. Evaluating such complex interventions effectively is not straightforward and so precise guidelines for clinicians are essential if small effects, consistent patterns of effects, non-specific effects and any ‘dose-response’ effects are to be documented systematically (see [41], and the accompanying article from Craig *et al*. [42], for guidance on the development and evaluation of complex interventions).

When asked how outcome was assessed (Question xii, Appendix 1), 36% of respondents reported using a structured interview, 53% use questionnaires, 16% use a VAS and just 4% use psychoacoustic measures at this stage (Fig. 1). The numbers of clinicians using a tinnitus-specific questionnaire to measure outcome was fewer than those using it for diagnosis. For example, the THI is used by 61 respondents (44%) in diagnosis but only 46 (34%) for outcome assessment (Fig. 2). No other tinnitus-specific questionnaires were reported for measuring outcome after management. Three individuals reported using the Client Oriented Scale of Improvement [43] to assess management outcome.

When asked about standardization of assessment procedures (Question xiii, Appendix 1) 38% reported standardization in their departments. However, a large proportion (28%) was unsure. As in the case of diagnostic assessment, we might again infer that there is the potential for different standards of care, given that patient benefit is not evaluated in a systematic way. No single management strategy is always effective in all patients and so management outcome should be monitored so that an alternative strategy can be prescribed if necessary.

### Determinants of a successful management outcome

We asked clinicians to describe what determines a good outcome (Question xiv, Appendix 1). The dominant theme was one of patient empowerment (stated in 38% of responses), to be achieved through patient education and their engagement with the therapeutic process. In total, 19% of respondents directly identify a holistic approach as key, requiring highly skilled audiologists and hearing therapists with a positive attitude to tinnitus management, working with a strong multi-disciplinary team. These are important aspects in the delivery of a complex health care intervention.

### Clinical skill in tinnitus care

Responses to Question xv (Appendix 1) indicated that 15% of audiologists and 90% of hearing therapists within the respective professions were considered to specialize in tinnitus. There are many routes to develop specialist clinical skills in tinnitus and so clinicians were asked about what type of training they had received (Questions xvi and xvii, Appendix 1). In total, 64% of respondents had attended the European Tinnitus Course (accredited by the British Society of Audiology), 57% had taken a Tinnitus Update Lecture Course (accredited by the British Society of Audiology), and 33% had undertaken the BTA tinnitus advisor training (accredited by the British Academy for Audiology and the British Society of Hearing Aid Audiologists). This latter 2-day course introduces participants' to the use of counselling skills in tinnitus care. More extensive training in the use of CBT is provided by Aston University (accredited by the British Academy of Audiology). In total, 31% of respondents had undertaken this training course.

It was reported that, within departments, 11% of all clinicians were trained in some form of counselling. However, 17% reported that their department had no staff trained in any form of counselling. When asked (Question xix, Appendix 1), 74% reported that they were in favour of audiologists undertaking additional training in psychological approaches to tinnitus management. A minority (<10%) expressed concerns that there is currently no evidence-base for the benefit, and that it should not be viewed as replacing the role of a clinical psychologist. The one clinical psychologist who responded welcomed audiologist training in psychological approaches to tinnitus management.

Overall, respondents are satisfied with the service they currently provide to tinnitus patients. A total of 70% considered their service to be effective. Only 4% described their service as ineffective. In total, 67% said they had sufficient resources to provide an effective tinnitus service. When asked about specific resource issues (Question xxi, Appendix 1), 17% identified a need for dedicated psychological support for tinnitus patients. A total of 16% felt that they had insufficient resources for training and equipment to supply an effective tinnitus service, whereas only 3% reported that they needed more time for the tinnitus patient. Given this, and the variability in clinical contact time offered by different respondents, the duration of consultations represents a potential target to improve the efficiency of the service, especially for those centres routinely offering appointments exceeding 1 hour.

## Discussion

This national survey describes how tinnitus patients are currently assessed and managed in audiology departments across England. While there are a number of points of agreement, not all practices are standardized. Almost all tinnitus patients are offered some ‘audiological’ management strategy such as a hearing aid or sound generator; however, the provision of psychological intervention is far more variable. This is of concern. The current lack of a cure or uniformly effective form of management for tinnitus, combined with what appears to be a low evidence-base for current practices implies that tinnitus care can be fragmented and cost ineffective [44].

With the exception of one, all respondents who reported their job role were audiologists or hearing therapists. It is worth noting therefore that training for audiological careers has undergone significant change in the last 10 years, the most recent (February 2010) being an extensive restructuring of UK audiology training [45]. Changes included the cessation of dedicated training programs in hearing therapy in 2004 (A. Casey, personnal communication) in favour of extended roles for audiologists through specialist training. We noted no significant differences between what was reported by hearing therapists and audiologists, with the exception that hearing therapists were significantly more likely to offer group sessions to tinnitus patients. There are two possible explanations for this finding, either that audiologists are sufficiently replacing the role of the hearing therapist, or that the declining numbers of hearing therapists has not yet reached the critical point at which an impact on care can be detected. It will be important in the future to closely monitor the care delivered to tinnitus patients given the unknown impact of major restructuring of audiological careers.

### Tinnitus assessment

Given the need to practice evidence-based health care, it is essential that clinicians pay attention to even small improvements in tinnitus intrusiveness, to accumulate evidence for the efficacy of current and potential tinnitus management strategies [46]. What resounds in the responses received to a number of questions, is that a person's tinnitus is very much appreciated to be as individual as the person themselves, and that this demands a holistic and tailored approach to assessment and management. However, patient reports do not provide an evidence-base unless they are collected in a systematic and repeatable way, that is, it is shared practice and appropriate evaluation of this practice that will provide the necessary evidence-base. The DH guidelines recommend that validated tinnitus questionnaires, the THI or the GHTQ, are used at the specialist centre level, but less than half of the clinicians we sampled ever use a validated tinnitus questionnaire. Crocetti *et al*. [47] found significant correlations between THI scores and scores for the same individuals on the BDI and State Trait Anxiety Inventory, recommending that assessments giving a THI score of 38 or more should be supplemented by a psychological consultation. The THI is therefore not only indicated as a useful direct measure of tinnitus but could also serve as an indicator of depression or anxiety co-morbidities. Only 7% of responders use a validated questionnaire for anxiety or depression, which is again concerning given that about 48% of a patient population are expected to present with a THI score above 38 [24]. Audiologists and hearing therapists clearly appreciate the psychological impact of tinnitus but the lack of validated measures (only one responder uses the BAI) makes inter-clinician reliability or best practice very difficult to measure, and weakens an argument for NHS commissioning of dedicated psychological support for tinnitus patients.

The GPG recommends the use of psychoacoustic measures such as tinnitus loudness to demonstrate stability of tinnitus to patients who may return to follow-up appointments complaining of increased tinnitus loudness. However, only 17% of respondents said that they recorded psychoacoustic measures of tinnitus at diagnostic appointments, and only 4% of respondents use psychoacoustic measures at follow-up. Why this recommendation receives so little use compared to self-reported measurements of tinnitus was not identified from the responses received but may reflect limited knowledge of the GPG. Anecdotal comments also suggest to us that there is little perceived clinical utility in psychoacoustic measures.

### Tinnitus management strategies

The major approaches to managing tinnitus are to compensate hearing loss, provide sound generators and provide directive and other forms of counselling. Quite how first-line management is decided may be directed by trial and error, the resources available or by personal judgements or patient preferences, implying unequal access to care. The consensus objective however is that of patient empowerment, through skilled education of the patient and their active engagement in the process of dealing with their tinnitus. Management is collaborative rather than prescriptive. Indeed, Henry *et al*. [48] identified this factor as important in effective self-management of tinnitus. Repeatedly respondents stated that their service for tinnitus patients is delivered on an individualized basis, that is, a patient-centered approach based on their interpretation of the patient's condition, level of understanding and expected ability to cope. However, to be able to call an intervention ‘patient-centred’, there is a need, again, to show evidence that it works in practice. Furthermore, a patient-centred approach is greatly enhanced when a structured model and tool is used [49]. No respondents referred to the use of a formal model of care or identified any tools for measuring patient readiness to engage in a programme of management.

The GPG does not constitute an evidence-base but rather recommendations from experts in the field. Consolidation of the existing evidence-base for DH recommended management strategies, in the form of systematic review or meta-analyses of published randomized-controlled trials would therefore be of benefit to clinicians and policy makers at this time. Sufficient evidence may already exist to further inform practice, for example, by indicating markers for deciding the first line of management for particular subsets of tinnitus patients.

### Conclusions

A lack of consensus in tinnitus services might provide flexibility to meet local demands, but it also has drawbacks; it is currently difficult to ascertain the key standards of best national practice for tinnitus, it makes the process of clinical audit (quality or cost-benefit) difficult, it affects equal patient access to treatments, and it will undoubtedly limit the speed of response to translational research outcomes, such as the speed of adopting new management strategies into clinical practice. Further to the GPG, we recommend the comprehensive use of tinnitus-specific validated self-reported measures in audiology departments at every appointment, as well as the standard use of anxiety and depression measures where indicated by the tinnitus measure scores, to identify those cases likely to benefit from counselling or onward referral to psychology. We further recommend a high-level review of the evidence for current management strategies and the subsequent development of guidelines that can be used to determine the first line of management offered to an individual, in a reproducible way. These will in turn allow evaluation of the management process towards best practice.

## Appendix 1

### Survey questions related to this report

- What is the typical duration of your tinnitus clinic appointments?
  Assessment only__ hour __ mins
  Treatment only__ hour __ mins
  Assessment and treatment combined__ hour __ mins
- Do you offer a tinnitus assessment and treatment in the same appointment?
  Yes__No__It depends (please specify __)
- Do you actively encourage family involvement?
  In the assessment stages
  In the treatment stages
  In the treatment outcome stages
- What sort of clinical appointments do you routinely offer?
  One-to-one with the individual
  Individual and a family member
  Group sessions (more than 2)
- What methods do you routinely use for tinnitus assessment?
  Audiogram
  Structured interview
  Unstructured interview
  Questionnaires (please specify __)
  Objective measures (e.g., tinnitus pitch and loudness)
  A visual analogue scale
- Do all tinnitus specialists in your ENT/Audiology service use the same assessment protocol? Yes__No__Not sure__
- In your view, how standardized should the tinnitus assessment procedure be?
- What formal treatments are offered at your ENT/Audiology service?
  Directive counselling (information and reassurance)
  Hearing aids
  Sound generators
  Tinnitus retraining therapy (or a version of)
  Stress management
  Cognitive-behavioural therapy
  Other form of psychological support (please specify __)
- Do you have the option to refer your tinnitus patients to a clinical psychologist or other specialist outside the ENT/Audiology service who is qualified in providing psychological therapy?
  Yes__No__
- What patient characteristics routinely determine your choice of treatment?
- Do you use different criteria for offering hearing aids to people who do or do not have tinnitus?Yes__No__
- What methods do you use for assessing treatment outcome?
  Structured interview
  Unstructured interview
  Questionnaires (please specify __)
  Objective measures (e.g., tinnitus pitch and loudness)
  A visual analogue scale
  Other (please specify __)
  None
- Is the assessment of treatment outcomes standardized across all tinnitus specialists in your ENT/Audiology service?
  Yes__No__Not sure__
- What do you consider to be important factors that determine a successful outcome from treatment?
- Please let us know how many different clinical specialists there are in your ENT/Audiology services that specialise in tinnitus:
  - Audiologist
  - Audiological physician
  - Hearing therapist
  - Audiological scientist
  - Audiological technician
  - ENT Consultant
  - Other (please specify)__
- What specialist tinnitus training have you received?
  European Tinnitus Course Tinnitus Update Lecture Course
  British Tinnitus Association lay-counselling for tinnitus course
  CBT in audiological practice (Aston University)
  Other (please specify __)
  None
- What conferences/lectures have you attended in the last 5 years?
  British Tinnitus Association annual conference
  British Academy of Audiology annual conference
  British Society of Audiology annual conference
  Other (please specify __)
  None
- How many individuals in your ENT/Audiology service are trained to provide counselling or other psychological support to people with tinnitus? __
- What is your view on audiologists undertaking additional training in psychological approaches to tinnitus management?
- In general, how effectively do you feel you are able to help people manage their tinnitus?
- Do you feel that you have access to sufficient resources to provide an effective tinnitus service?
- Your job title:
